# Probable sleep bruxism in children in the mixed dentition stage: prevalence and association with the risk for obstructive sleep apnea—a cross-sectional study

**DOI:** 10.3389/froh.2026.1872176

**Published:** 2026-07-21

**Authors:** Joseph Hajjar, Amine El Zoghbi, Angela Nader, Lara Nasr, Nada R. Chedid

**Affiliations:** 1 Department of Pediatric Dentistry, Faculty of Dental Medicine, Saint Joseph University of Beirut, Beirut, Lebanon; 2 Department of Prosthodontics and Occlusion, Faculty of Dental Medicine, Saint Joseph University of Beirut, Beirut, Lebanon; 3 Cranio-Facial Research Laboratory, Faculty of Dental Medicine, Saint Joseph University of Beirut, Beirut, Lebanon

**Keywords:** cross-sectional study, mixed dentition, obstructive sleep apnea, pediatric dentistry, sleep bruxism

## Abstract

**Background and aim:**

Sleep bruxism's (SB) association with obstructive sleep apnea (OSA) is still not conclusively established. The present study aimed to assess the prevalence of probable sleep bruxism (PSB) in a sample of Lebanese children aged six to ten years, and evaluate the association between PSB and OSA risk.

**Materials and methods:**

Children recruited from schools were examined for PSB's intra-oral signs, occlusal and mucosal factors. Parents/guardians filled one survey addressing their child's anamnesis, socio-demographic and daily habits' information and another [Pediatric Sleep Questionnaire (PSQ)] evaluating their OSA risk. Chi-square, Mann–Whitney and logistic regression tests were run between PSB and independent variables. Significance level was set at 0.05.

**Results:**

Among 281 participants, 61.2% showed PSB. PSQ score and the percentage of patients at risk of OSA were higher among PSB than non-PSB patients, without statistical significance (*p* = 0.169 & 0.238 respectively). Logistic regression confirmed a clear yet non-significant association between PSB and PSQ score (*p* = 0.058).

**Conclusion:**

PSB was identified in 61.2% of the study sample. A positive yet non-significant association was found between PSB and OSA-risk variables. Further investigations are needed to better understand this association.

## Introduction

Sleep bruxism (SB) is defined as involuntary, episodic, and repetitive jaw muscle activity, accompanied by teeth clenching or grinding and mandible's bracing or thrusting (1). This multifactorial condition affecting adults and children is influenced by central factors, such as emotional stress, or morphologic factors such as occlusal interferences (2). Prevalence ranges between 1% and 15% in adults and between 3% and 49% (2) in children and adolescents. This variability may be ascribed to two main reasons: different sleep bruxism definitions and different age groups (3) in previous studies. Lobbezoo et al. (4) graded SB as follows: possible SB, for which diagnosis relies upon one of the guardians/parents reporting some noise or sounds of tooth grinding during the patient's sleep; probable SB, diagnosed besides the above-cited report by clinical features of bruxism noted during the functional examination, such as increased tooth wear, masticatory muscle pain or fatigue and muscle hypertrophy; and definite SB, where final diagnosis is based on the gold standard tool, polysomnography (PSG), in adjunction to tooth grinding sounds’ report and SB's clinical features' depiction (4).

PSG use in pediatric patients is complex and time-consuming, thus difficult (1). Therefore probable sleep bruxism (PSB) is currently considered the most appropriate way to assess pediatric SB, relying on detected wear on primary and permanent teeth, in addition to parents recounting SB sounds or symptoms reportedly experienced by their children (5). PSB prevalence is higher in the mixed than in the primary dentition (6), with SB risk factors for the former discussed in previous studies, including occlusal factors, psychological factors, socio-demographic factors and pathophysiological factors such as Gastro-esophageal reflux disorder (GERD) and sleep-related breathing disorders (SRBDs) (1, 7). SRBDs are a range of conditions during sleep, ranging from a habitual snoring through upper airway resistance to obstructive sleep apnea (OSA) syndrome, a multifactorial disease with significant heterogeneity in presentation and co-morbidities. Its prevalence in children ranges approximately between 1% and 4% using objective measures (8). Children diagnosed with OSA may suffer from attention deficit, learning delay, memory consolidation impairment, aggressive behaviors, metabolic disorders, and cardiovascular diseases, which can worsen in adulthood (9). Attempts made to establish an association between SB and high OSA risk in adults and children were inconclusive (10, 11).

There is renewed interest towards pediatric SB and its possible association to sleep breathing disorders (12), however such investigations lack in Lebanon as far as the present article's authors know. Therefore, the present study's primary objective was to assess the PSB's prevalence in a sample of Lebanese children in the mixed dentition. A secondary objective aimed to evaluate the association between OSA risk and PSB in the study population. Such assessment may be useful to the future establishment of applicable diagnostic guidelines for dental clinicians to depict sleep apnea's intra-oral signs.

## Material and methods

The present study received approval from the Ethics Committee of Saint Joseph University of Beirut*,* Lebanon (certificate number: 2023-201). Parents consented to their respective children's participation by signing an informed consent statement.

### Study design and sample selection

The study's population was selected from schools in Beirut and Mount Lebanon, districts that gather a considerable proportion of Lebanese children. Using the Epi Info^TM^ software, and considering a current population size of 550,000 Lebanese children aged between six and ten years (according to the Lebanese Central Administration of Statistics, 2023), the minimum sample size calculated was 127 individuals with an expected prevalence of PSB of 9.1% (13), a confidence level of 95% and a 5% acceptable margin of error.

Included participants fulfilled the following criteria:

- aged six to ten years, enrolled in a school
- healthy (ASA I) or having a well-controlled disease without any functional limitation (ASA II), nor any healthcare special need
- having at least one primary tooth in each quadrant and all first permanent molars in occlusion
- with centric occlusion corresponding to maximum intercuspation.

Patients excluded from the study were:

- aged less than six or more than 10 years
- presenting first permanent molars not in occlusion yet
- displaying premature contact between upper and lower primary teeth
- currently undergoing orthodontic or ortho-interceptive treatment
- presenting with either a complete permanent or primary dentition
- presenting with special healthcare needs
- presenting with severe, possibly life-threatening systemic conditions
- returning incompletely filled questionnaires.

### Data collection

Data collection was accomplished by administering two questionnaires to parents/guardians, followed by a clinical examination of the children performed by two trained and calibrated observers (14).

#### Questionnaires

The first questionnaire sought information about the family's socio-economic status, the child's age, gender, general health status and the presence of harmful oral habits. Additionally, it collected information as reported by the parents concerning sounds of tooth grinding or tooth clenching associated with bruxism during sleep, jaw muscle discomfort and morning headaches reportedly experienced by the child during the three months preceding the examination date (4).

Parents/guardians were also addressed the Pediatric Sleep Questionnaire (PSQ), a 22-items validated tool used as an alternative to the PSG for OSA's risk assessment in pediatric patients; sensitivity and specificity were 0.85 and 0.87 respectively (15). Possible responses for all items are either Yes = 1, No = 0, or Don't know = Missing. The final cumulative score (PSQ score) was calculated from the Yes and No responses only with a cut-off of 0.33, indicating the risk of sleep-disorder breathing, meaning that 33% of the questionnaire items were answered positively. PSQ's psychometric properties are considered acceptable to assess OSA risk in children (15), which makes it one of the most widely used screening tools for OSA moderate-to-severe risk assessment in pediatric populations, especially in field-based studies (16). A license for the use of the PSQ's original version was obtained from the University of Michigan. An Arabic version cross-culturally validated in 2023 was used in the present study with the consent of its author (17).

#### Clinical assessment

Patients whose parents answered the questionnaires underwent a clinical oral cavity examination assessing tooth wear, anterior crossbite, posterior crossbite and signs of lips/cheeks/tongue bite. The examination took place on site at each selected school, after the children brushed their teeth, in a room illuminated by ceiling lighting, with each child seated on a regular chair. Teeth were kept moist with excess saliva removed using gauze pads, and examined using a flat mouth mirror and a tongue depressor, under a pen-light intra-oral illumination. Tooth wear was determined according to Smith and Knight's index (18) for occlusal surfaces on a tooth-by-tooth basis:

➣ 0 = no loss of enamel surface characteristics
➣ 1 = loss of enamel surface characteristics
➣ 2 = loss of enamel exposing dentine on less than one-third of surface
➣ 3 = loss of enamel exposing dentine on more than one-third of surface
➣ 4 = complete enamel loss—pulp exposure—secondary dentine exposure

Tooth wear was recorded when a tooth displayed a score of 1–4.

PSB's diagnosis, which was the main outcome of the present study, relied upon positive findings of tooth attrition wear, with or without parents/guardians's report (4). Tooth wear was confirmed when at least three teeth [primary molars and/or canines with or without permanent molar(s)] located in different quadrants (Figure 1) manifested occlusal/incisal wear, according to Wetselaar and Lobbezoo's guideline of tooth wear evaluation (5), and similarly to the studies conducted by Drumond et al. (19) and Mota-Veloso et al.’s (20). Anterior and posterior crossbites were assessed because of their association with mouth breathing, the latter also associated with pediatric sleep bruxism (21). Mucosal signs of cheeks, lips and/or tongue biting, such as linea alba and/or teeth indentations on the lips or the tongue's border were clinically evaluated (4). Intra-oral frontal and lateral pictures documented tooth wear and occlusion in the transverse and sagittal planes. Inter-examiner agreement was evaluated between the two observers by Cohan Kappa's coefficient; the values were 0.89, 0.924, 0.946 and 0.849 for the clinical confirmation of tooth wear, posterior crossbite, anterior crossbite and cheeks, lips and/or tongue biting respectively, indicating an excellent level of agreement (14).

**Figure 1:**
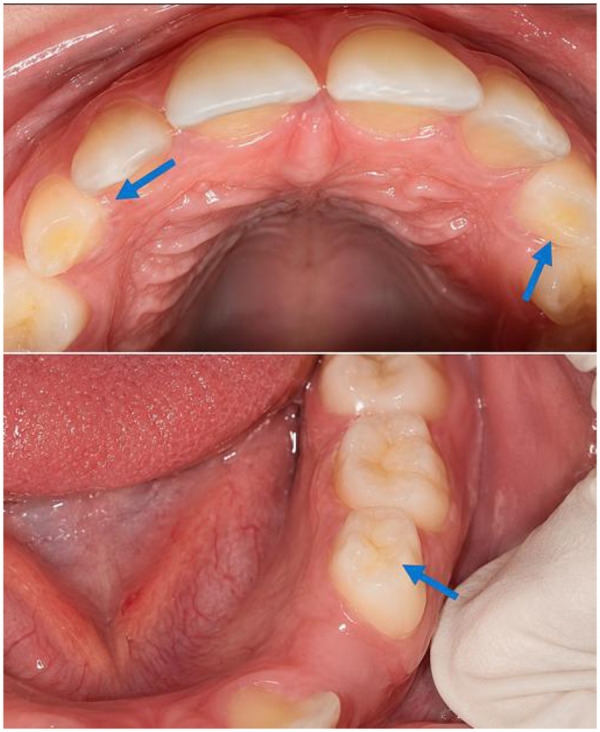
Example of a case from the study sample where a minimum of three worn teeth in three different quadrants are detected upon clinical examination.

### Statistical analysis

Data analysis was performed using SPSS® Statistics for Windows (Version 23) (IBM Corp., Armonk, NY, USA) with a 95% confidence interval and a 0.05 significance level. In terms of descriptive statistics, medians and interquartile were reported for quantitative variables, while frequencies and percentages were reported for qualitative ones. Bivariate analysis consisted of the *U*-Mann–Whtiney and Chi-square tests to assess the association between the presence of PSB and continuous and categorical variables respectively. Multivariate analysis used the logistic regression test, including all variables for which association with PSB was determined by a *p*-value ≤0.2, to obtain the adjusted odds ratio and assess the true association between PSB occurrence and PSQ score, while attenuating the confounding effect of co-variables.

## Results

### Descriptive statistics

Out of 696 assigned questionnaires, 345 completed questionnaires were returned (response rate 49.56%), and the corresponding participants were clinically assessed. 64 participants were further excluded for reasons related to the inclusion/exclusion criteria, including 32 participants with missing data; no imputation procedures were performed. The remaining ones did not match the inclusion criteria. This led to 281 eligible subjects having undergone the clinical examination (Figure 2).

**Figure 2:**
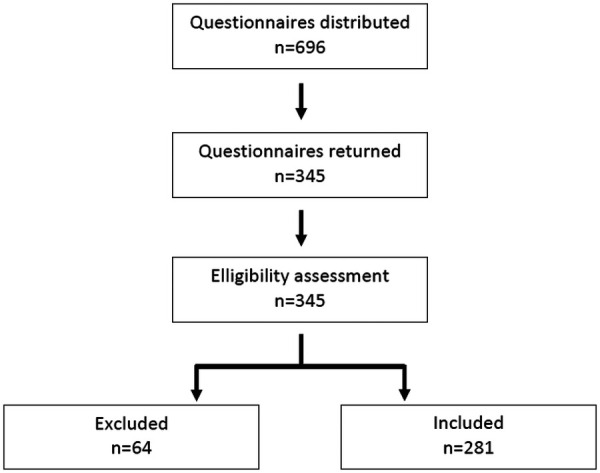
Flowchart of participant recruitment, eligibility assessment, exclusions, and final inclusion in the study.

Participants with PSB represented 61.2% (Table 1, Figure 3), however, 20.3% of parents/guardians reported PSB signs in their children. On a socio-demographic level, the sample's median age was 9 ± 2 years with 50.5% female participants. Only 17.4% reported current health conditions (asthma, allergic rhinitis, gastro-esophageal reflux disorder, ADHD, pneumonia … ). On a socio-economic level, 72.2% of mothers had university degrees, while 51.2% of mothers were working outside their homes. 51.8% of families included four members or less residing at the home and 54.8% included one or two children. Not more than two siblings were sharing a room in 79.4% of participants' corresponding families. In terms of birth order, 47.3% were the eldest among their siblings, while 31.3% were second in birth order; 21.4% of children were the third or younger among their siblings. Biting nails, objects and lips/cheeks/tongue was reported in the sample occasionally (answer: “sometimes”) or frequently (answer: “yes”) with the respective percentages 32.7, 22.1 and 17.5. Upon oral examination, clinical signs of cheeks/lips/tongue bite, posterior crossbite and anterior crossbite were present in 5%, 8.9% and 13.5% of participants respectively. The median PSQ score was 0.09 (IQR: 0.18). Considering a cut-off of 0.33 to confirm OSA risk's presence in children (15), 7.1% presented this risk.

**Table 1:** Descriptive display of all assessed variable in the study.

Variables	*N* = 281
Age^†^	9 (2)
Sex	
Male^‡^	139 (49.5)
Female^‡^	142 (50.5)
Health condition	
Present^‡^	49 (17.4)
Absent^‡^	232 (82.6)
Mother's schooling	
School degrees (primary, brevet, baccalaureate)^‡^	78 (27.8)
University bachelor and post-graduate degrees^‡^	203 (72.2)
Mother works outside home	
Yes^‡^	144 (51.2)
No^‡^	137 (48.8)
Number of residents at home	
≤4^‡^	145 (51.6)
>4^‡^	136 (48.4)
Number of children	
≤2^‡^	154 (54.8)
>2^‡^	127 (45.2)
Number of siblings sharing the same room	
≤2^‡^	223 (79.4)
>2^‡^	58 (20.6)
Birth order	
First^‡^	133 (47.3)
Second^‡^	88 (31.3)
Younger than the 2nd^‡^	60 (21.4)
Nail biting	
Present^‡^	92 (32.7)
Absent^‡^	189 (67.3)
Object's biting	
Present^‡^	62 (22.1)
Absent^‡^	219 (77.9)
Lips, tongue, cheek's biting (parents' report)	
Present^‡^	49 (17.5)
Absent^‡^	232 (82.6)
Clinical signs of lips, tongue, and/or cheek's biting	
Present^‡^	14 (5)
Absent^‡^	267 (95)
Posterior crossbite	
Present^‡^	25 (8.9)
Absent^‡^	256 (91.1)
Anterior crossbite	
Present^‡^	38 (13.5)
Absent^‡^	243 (86.5)
PSQ Score^†^	0.09 (0.18)
Risk for OSA	
Present (PSQ ≥0.33)^‡^	20 (7.1)
Absent (PSQ <0.33)^‡^	261 (92.9)
PSB (clinical signs)	
Present^‡^	172 (61.2)
Absent^‡^	109 (38.8)
Parents/guardians report of signs of bruxism	
Present^‡^	57 (20.3)
Absent^‡^	224 (79.3)

PSQ: pediatric sleep questionnaire; OSA: obstructive sleep apnea; PSB: probable sleep bruxism.

† Median (Interquartile).

‡ Number (%).

**Figure 3:**
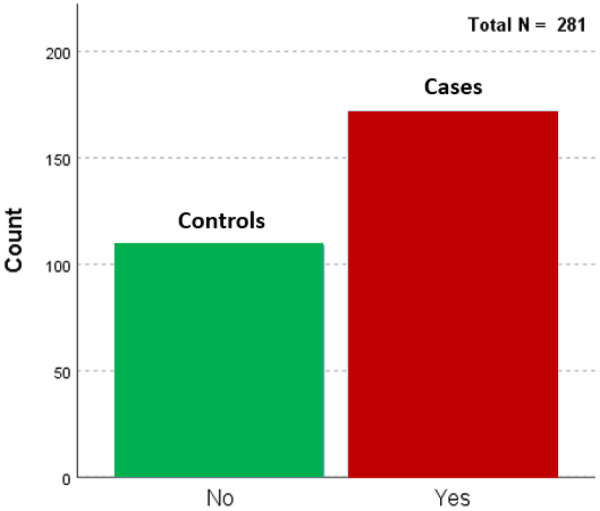
Comparison between the proportion of participants diagnosed with PSB (cases) with those who do not manifest this condition (controls).

### Analytical statistics

Chi-square test was run between PSB's presence and all categorical risk factors (Table 2). No statistically significant difference between PSB groups were shown among all these variables, except for the presence of anterior crossbite, where *p* was 0.012 (<0.05). A higher percentage of patients presenting with anterior crossbite was found in children without PSB (20.2%) in comparison to those with PSB (9.3%). Regarding OSA risk, fifteen bruxers (8.4%) were at risk of OSA, while five non-bruxers (4.7%) had the same risk. Despite these differences, the association between PSB and OSA risk was not statistically significant (*p*-value = 0.283 > 0.05). For some variables related to socio-economic status (number of residents at home and number of siblings sharing a room in the home), there were slight non-statistically significant differences between PSB groups (*p* = 0.098 and 0.096 respectively). Children from families with more than four residents in the home presented PSB (52.3%) more than those including four residents or less (42.2%). Moreover, when the number of siblings sharing a room exceeded two, a higher proportion of PSB was found (23.8% in PSB vs. 15.6% in non-PSB).

**Table 2:** Chi-square analysis between the occurrence of probable sleep bruxism and all categorical risk factors.

Variables	*N* = 281		*p*-value
	PSB		
	Present (*n* = 172)	Absent (*n* = 109)	
	*n* (%)		
Gender			0.903
Male	86 (50)	53 (48.6)	
Female	86 (50)	56 (51.4)	
Health condition			0.872
Present	29 (16.9)	20 (18.3)	
Absent	143 (83.1)	89 (81.7)	
Mother schooling			0.537
School degree	50 (29.1)	28 (25.7)	
University degree	122 (70.9)	81 (74.3)	
Number of residents at home			0.098
≤4	82 (47.7)	63 (57.8)	
>4	90 (52.3)	46 (42.2)	
Birth order			0.094
The eldest	77 (44.8)	56 (51.4)	
The second	51 (29.7)	37 (33.9)	
Younger	44 (25.6)	16 (14.7)	
Number of siblings sharing the same room			0.096
≤2	131 (76.2)	92 (84.4)	
>2	41 (23.8)	17 (15.6)	
Number of children in the family			0.294
≤2	90 (52.3)	64 (58.7)	
>2	82 (47.7)	45 (41.3)	
Mother works outside home			0.329
Yes	84 (48.8)	60 (55)	
No	88 (51.2)	49 (45)	
Nail biting			0.98
Present	57 (33.1)	35 (32.1)	
Absent	115 (66.9)	74 (67.9)	
Object's biting			0.774
Present	35 (20.3)	27 (24.7)	
Absent	137 (79.7)	82 (75.2)	
Lips, tongue, cheek biting			0.704
Present	28 (16.3)	21 (19.3)	
Absent	144 (83.7)	88 (80.7)	
Clinical signs of Lips/tongue/cheeks bite			0.783
Present	8 (4.7)	6 (5.5)	
Absent	164 (95.3)	103 (94.5)	
Posterior crossbite			1
Present	15 (8.7)	10 (9.2)	
Absent	157 (91.3)	99 (90.8)	
Anterior crossbite			0.012*
Present	16 (9.3)	22 (20.2)	
Absent	156 (90.7)	87 (79.8)	
OSA risk			0.238
Absent (less than 0.33)	157 (91.3)	104 (95.4)	
Present (0.33 and more)	15 (8.7)	5 (4.6)	
Parents' report of signs of sleep bruxism			1
Present	35 (20.3)	22 (20.2)	
Absent	137 (79.7)	87 (79.8)	

PSB: probable sleep bruxism; N: study sample size; n: frequency; %: valid percentage; OSA: obstructive sleep apnea.

* *P*-value <0.05 is significant.

The Mann–Whitney test was run out between presence of PSB and age and PSQ score (Table 3). No statistically significant difference between bruxers and non-bruxers was shown (*p*-values = 0.184 and 0.169 > 0.05, respectively). However, clinical differences were found in the PSQ score's medians between bruxers and non-bruxers (0.16 vs. 0.05, respectively) (Figure 4).

**Table 3:** Mann–Whitney analysis between the presence of PSB and the age and PSQ score.

Variables	*N* = 281		*p*-value
	PSB		
	Present (*n* = 172)	Absent (*n* = 109)	
Age^†^	9 (2)	9 (2)	0.184
PSQ Score^†^	0.16 (0.19)	0.05 (0.136)	0.169

PSB: probable sleep bruxism; PSQ score: Pediatric Sleep Questionnaire score.

† Median (Interquartile).

*P*-value <0.05 is significant.

**Figure 4:**
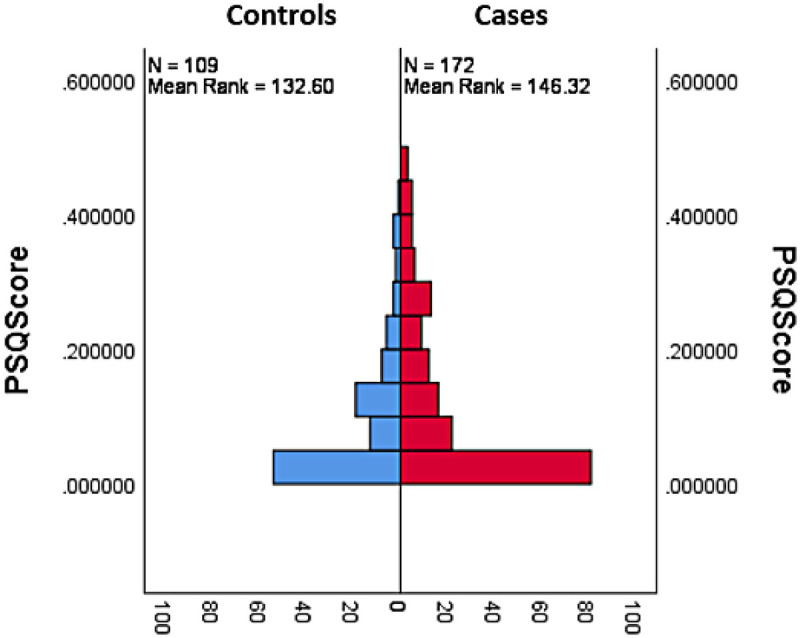
Display of the difference between cases and controls in terms of the variation of the PSQ score across the study sample, according to the *U* Mann–Whitney test.

A binary logistic regression analysis was conducted to predict PSB using all variables for which corresponding *p*-values were less than 0.2. Anterior crossbite had a statistically significant association with PSB (*p*-value = 0.02), while no statistically significant difference was shown between PSB groups among the other variables, including PSQ score, despite its corresponding *p*-value (0.07) close to the significance level (Table 4).

**Table 4:** Logistic regression for the assessment of the accurate relationship between the presence of PSB and the PSQ score.

Risk factor	PSB		Unadjusted OR	Adjusted OR	CI 95% for the adjusted OR		*p*-value
	Present	Absent			Lower	Upper	
PSQ score^†^	0.16 (0.19)	0.05 (0.136)	2.142	8.512	0.845	74.642	0.07
Anterior crossbite^‡^	16 (9.3)	22 (20.2)	−0.85	0.427	0.209	0.876	0.02*

PSB: probable sleep bruxism; PSQ: Pediatric Sleep Questionnaire; OR: odds ratio; CI: confidence interval.

† Median (Interquartile).

‡ Number (%).

* *P*-value <0.05 is significant.

## Discussion

The proportion of PSB found in the present study (61.2%) exceeds those of previous studies in the mixed dentition, ranging from 9.1% to 40% (17, 22–25), particularly the prevalence (9.1%) in Leal et al.'s study (13), where a comparable methodology and similar PSB diagnostic criteria were applied on a similar mean age sample.

According to Lobbezoo et al. (23), pediatric PSB's diagnosis should rely both on parental reports and positive clinical findings suggestive of SB. However, this consensus' updated 2017 version suggested PSB's diagnosis could rely upon clinical findings with or without parental report of SB signs in children (4). Despite no final agreement so far, some studies based their PSB's diagnosis on a minimum of three teeth displaying tooth wear (17, 26). In the present study, in addition to the latter criterion, diagnostic teeth were located in at least three different quadrants, suggesting generalized bruxism (5) (Figure 5), to rule out teeth interference's related grinding. Parental report was not considered for PSB diagnosis because of its low accuracy (4, 24). Therefore, prevalence differences between this study and Leal et al.’s is not due to significant methodological differences.

**Figure 5:**
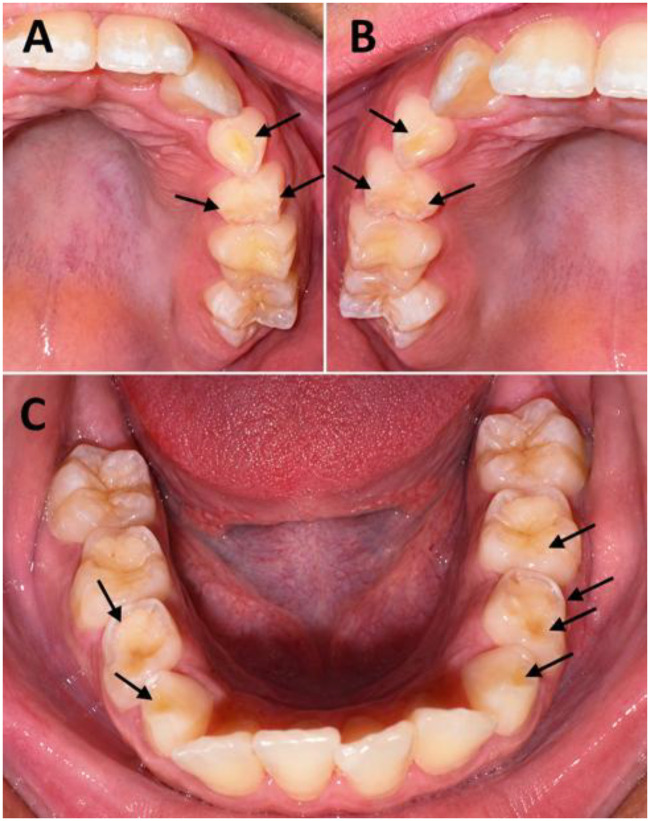
10-year-old-girl from the study sample displaying generalized tooth wear in the maxillary left **(A)** and right **(B)** molar quadrants, and mandibular right and left quadrants **(C)** (black arrows marking specific wear sites).

Since no diagnostic instruments were used, tooth wear's clinical signs weren't necessarily associated with bruxism occurring at the time when the children were examined. Some wear patterns could have come along with normal development resulting in a physiological tooth grinding phenomenon in children during sleep (1). Therefore, these signs could have appeared earlier during previous bruxism episodes, justifying the label of “probable” vs. “definite” sleep bruxism, the latter being diagnosed through PSG with simultaneous audio/video recordings of patients (4). In children, the use of PSG is not readily feasible because of their low tolerance and the handling of these devices. The instrumental approach needs a minimum level of compliance and cooperation for measurements' higher accuracy and reproducibility. Therefore using a more standardized and elaborate tool addressing all aspects of the patient's anamnesis (familial, genetic, psychosocial, medical, behavioral, dental, occlusal … ) is suggested to assess bruxism's occurrence and increase the proposed diagnosis' accuracy, such as Manfredini et al.'s recently (2023) elaborated Standardized Tool for the Assessment of Bruxism (STAB), a two axes tool including 14 domains, for a total of 66 items which may need further refinement and adaptation for the pediatric population (25).

The variation could also be attributed to sample size differences between studies (281 vs. 739) and the possibility that the present study may not be representative of the whole Lebanese population, with a potential selection bias.

Stress and anxiety are potential risk factors for pediatric SB, since bruxism might be a way to activate the parasympathetic braking system, to counteract sympathetic activity in high-stress and anxiety-inducing situations (27), leading to the unconscious activation of the masticatory muscles through the central nervous system commands during sleep (28).

The high PSB percentage could therefore be likely allocated to high stress levels experienced by children during the past five years in Lebanon, a low-middle income Middle Eastern nation. During this period, an economic crisis of unprecedented proportions started in 2019, concomitantly with the coronavirus infectious disease (COVID-19), declared a pandemic in march 2020 by the World Health Organization (WHO) (29). The pandemic itself negatively impacted people's mental health and successive WHO-recommended lockdowns worsened the already shaky Lebanese socio-economic situation. The consequent sudden decrease in socio-economic status and stressful events accompanying the pandemic increased parental stress and anxiety levels, leading to higher such levels in their children (30). Lockdowns along with lifestyle changes because of subsequent social restrictions and schools' closure also triggered in many children the development of fear, nervousness, depression and anxiety, decreased physical activity, increased screen exposure, unhealthy diet, loneliness …  (31). Moreover, a higher prevalence of moderate to severe food insecurity in Lebanon, defined as the lack of access to nutritious healthy food due to economic and financial insufficiencies, was found by Gedeon et al. (32) as a result of the COVID pandemic and socio-economic crisis. Higher levels and longer-lasting phases of food insecurity lead to decreased mental health outcomes among children (32, 33).

The effect of lower socio-economic status on increasing bruxism risk was found in the present study, since an increase in the number of residents in the home (*p*-value = 0.098) and the number of siblings sharing a room (*p*-value = 0.096) showed a slightly higher, although non-significant, PSB percentage.

In the present study, parents were asked to report whether they noticed nocturnal sounds or morning signs reportedly felt by their children and suggestive of SB, to determine whether parents/guardians are aware of SB's occurrence in their children. Table 2 shows no association between PSB and parents' report (*p*-value = 1), which may mean that parents are poorly aware of their children's bruxism during sleep and of risk factors behind SB, such as OSA (9, 34, 35), underdiagnosed in the pediatric population. More parental awareness is therefore required to shed light on bruxism and possible related comorbidities among Lebanese children, and pediatric dentists can play an important role in raising such awareness.

The secondary objective of the present study was to assess the relationship between PSB and OSA risk. A higher PSB percentage was found among patients at risk of OSA (Table 2) while a higher PSQ score emerged among PSB patients (Table 4). Although no statistical significance was found, results suggest a possible relationship between PSB and OSA risk, also found by Tachibana et al. (9), and explained by BornHardt et al. (36) by the fact that subsequent to apnea/hyperpnea events, lower oxygen levels reaching the brain might trigger activation of the genioglossus and masseter muscles, that stabilize the forward mandibular position, and prompt the position of the hyoid bone in order to preserve the upper airway lumen's patency. Moreover, respiratory events (hypopnea/apnea) due to OSA lead to sleep arousals which provoke rhythmic masticatory muscles' activity and therefore SB (36). Further studies on a larger sample size may be needed for more significant results.

The non-significant association between bruxism and gender (*p*-value = 0.903) (Table 2) replicates previous studies' results (6, 13, 19). Nail and objects' biting were not significantly associated with PSB in the present study (Table 2), unlike in Drumond et al.'s study (19). These factors are related to stress, a potential risk factor for PSB as mentioned before; non-significance in the present study may be related to the small sample size. Posterior crossbite and PSB in the present study were non-significantly related, (*p*-value = 1) as in Lins et al.'s (16) where a non-significant relationship between PSB and anterior crossbite was found. However, in the present study significantly more anterior crossbite cases were found among non-PSB (20.2%) vs. PSB patients (9.3%) (*p*-value = 0.02) (Table 4), whereas no relationship between SB and anterior crossbite was previously described in the literature. This finding deserves more investigation, and should be interpreted with caution, because it may be an incidental observation or influenced by unmeasured craniofacial variables. Further research could be led taking into account additional occlusal factors like Angle occlusion's class and the degree of anterior crossbite [involved teeth's number, crossbite's upper or lower origin (37) … ]. All other independent variables did not impact PSB, with no significant associations found.

To the authors' knowledge, this is the first study evaluating probable sleep bruxism and its association with OSA risk in Lebanese children using a validated Arabic version of the Pediatric Sleep Questionnaire, aligning with Lebanon's national language. The present study's findings may help initiate parents and dentists, particularly pediatric dentists, to SB occurrence among Lebanese children, increasing awareness of pediatric SB and its possible risk factors. Some dental clinicians consider SB in pediatric patients as only a physiologic process favoring teeth growth and eruption, however it is a very complex condition with many possible underlying risk factors including several comorbidities like OSA (36). Consequently, there may be more pronounced wear on primary teeth, with more severe dental substance loss (Figure 6), suggestive of a higher than physiologically normal bruxism level. The present study's relevance resides in that pediatric SB diagnosis could serve in early OSA risk assessment in children, and its subsequent possible diagnosis, helping to avoid the latter's harmful impact (9). Moreover, PSB's diagnosis in children can be an indicator of their cooperation level in the dental care setting. A higher PSB percentage could suggest higher stress and anxiety levels, which can be extrapolated to affect the child's behavior at the dentist's. Hence, this condition's management should be multidisciplinary, including all the healthcare professionals needed to address its different aspects (psychology, physiopathology, occlusal … ) in children.

**Figure 6:**
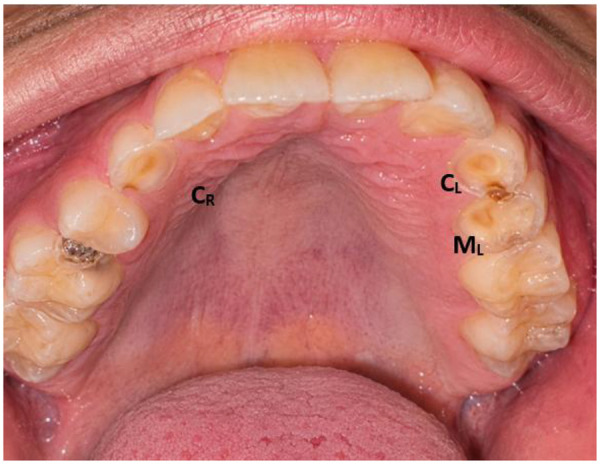
Severe wear displayed on upper primary right (*C_R_*) and left (*C_L_*) canines, and upper left primary first molar (M) in an nine-year-old boy from the study sample.

The present study's limitations include the cross-sectional design that, despite choosing Lebanon's two most populated districts, may not be representative of all Lebanese areas, and the relatively small sample size. Besides, an information bias may have resulted from a certain amount of gathered data being based upon parents/guardians' answers to the questionnaires. Some of the collected information could be inaccurately or untruthfully reported by the respondents, including data about families' socio-economic status and children's general health; of particular concern is the accurate reporting of ADHD: some parents could have been aware of its presence without mentioning it. This possible bias applies for the PSQ as well, which may have led to the non-significant association found between PSB and OSA risk. On the other hand, pediatric OSA is multifactorial; and the use of the PSQ to assess OSA risk in children bypassed several craniofacial and upper airway characteristics, including skeletal discrepancies, adenotonsillar hypertrophy, and upper airway dimensions, which are potential risk factors for pediatric OSA (8). These factors were not assessed in the present study and may have influenced both the estimated OSA risk and its association with probable sleep bruxism. Moreover, the diagnosis of PSB was largely based upon the clinical depiction of tooth wear. Although efforts were made to identify wear patterns compatible with attrition, distinguishing clinically between attrition, abrasion, erosion, or multifactorial wear remains challenging in epidemiological field-based settings. In consequence, some lesions attributed to PSB may have been influenced by other etiological factors, such as physiological wear, some parafunctional habits unrelated to sleep bruxism (nail and objects biting, awake bruxism … ), dietary erosive factors or GERD-related erosions …  (7) potentially affecting the prevalence estimates reported in the present study.

In conclusion, the present cross-sectional study aimed to assess PSB's prevalence in a sample of Lebanese 6-to-10-year-old children in the mixed dentition stage and found it to be 61.2%. This relatively high percentage may be attributed to the diagnostic method for PSB based on clinical examination of tooth wear, and not on actual observation of SB occurrence episodes, and to the ongoing Lebanese economic crisis, which occurred simultaneously with the COVID-19 pandemic and caused very high stress levels among parents and children. A higher although statistically non-significant risk for OSA was found among bruxer children, implying the need for further investigations. Moreover, a significant relationship between PSB and anterior crossbite was found, with a higher prevalence of the latter among non-bruxer children, without explanations found in the literature so far. Further longitudinal comparative randomized studies conducted on larger samples with a standardized tool for assessing pediatric SB should be performed to ascertain the present study's results.

## Data Availability

The raw data supporting the conclusions of this article will be made available by the authors, without undue reservation.
